# Enzyme Assembly for Compartmentalized Metabolic Flux Control

**DOI:** 10.3390/metabo10040125

**Published:** 2020-03-26

**Authors:** Xueqin Lv, Shixiu Cui, Yang Gu, Jianghua Li, Guocheng Du, Long Liu

**Affiliations:** 1 Key Laboratory of Carbohydrate Chemistry and Biotechnology, Ministry of Education, Jiangnan University, Wuxi 214122, China; lvxueqin@jiangnan.edu.cn (X.L.); cuishixiu@vip.jiangnan.edu.cn (S.C.); guyang@vip.jiangnan.edu.cn (Y.G.); lijianghua@jiangnan.edu.cn (J.L.); gcdu@jiangnan.edu.cn (G.D.); 2 Key Laboratory of Industrial Biotechnology, Ministry of Education, Jiangnan University, Wuxi 214122, China

**Keywords:** enzyme assembly, scaffold-free assembly, artificial scaffold, physical compartment, metabolic flux

## Abstract

Enzyme assembly by ligand binding or physically sequestrating enzymes, substrates, or metabolites into isolated compartments can bring key molecules closer to enhance the flux of a metabolic pathway. The emergence of enzyme assembly has provided both opportunities and challenges for metabolic engineering. At present, with the development of synthetic biology and systems biology, a variety of enzyme assembly strategies have been proposed, from the initial direct enzyme fusion to scaffold-free assembly, as well as artificial scaffolds, such as nucleic acid/protein scaffolds, and even some more complex physical compartments. These assembly strategies have been explored and applied to the synthesis of various important bio-based products, and have achieved different degrees of success. Despite some achievements, enzyme assembly, especially in vivo, still has many problems that have attracted significant attention from researchers. Here, we focus on some selected examples to review recent research on scaffold-free strategies, synthetic artificial scaffolds, and physical compartments for enzyme assembly or pathway sequestration, and we discuss their notable advances. In addition, the potential applications and challenges in the applications are highlighted.

## 1. Introduction

As a potential alternative to synthetic chemistry, metabolic engineering aims to enhance the production of native metabolites or to endow cells with the ability to produce new products [1]. To achieve this aim, various strategies have been proposed and developed, including the construction of novel pathways out of enzymes from different organisms [2], synthetic sRNAs to identify and modulate the expression of target genes [3], global optimization of the metabolic network through module engineering [4,5], and dynamic regulation or change in the intracellular metabolic flow direction through dynamic metabolic engineering [6,7]. However, it is still challenging to use these strategies to achieve efficient commercial production, due to flux imbalances or low enzymatic activities [8]. This is especially the case for exogenous synthesis pathways, because the overproduction of desirable products is often unfavorable or unnecessary for the survival or normal growth of the host, and with the increasing complexity of the synthesis pathway, problems such as the accumulation of toxic metabolites or undesirable cross-talk with other cell pathways are introduced [9,10,11]. Therefore, reducing these limitations to improve the output of desired products is the key problem to be solved urgently in metabolic engineering.

To combat these issues, the construction of multi-protein supramolecular systems is of broad interest, owing to its potential applications in nanotechnology, synthetic biology, systems biology, and multienzyme biocatalysis [12,13]. Inspired by natural multi-enzyme complexes, such as the AROM complex and the tryptophan synthase α_2_β_2_ complex [14,15], synthetic biologists seek to achieve the spatial enzyme organization of heterogenous metabolic pathways to improve the output of high-value products for industrial and pharmaceutical applications [16,17]. Through a series of methods and strategies, such as metabolomics and kinetic model building, researchers found that enzyme assembly can control the local concentrations of reactants, intermediates, and enzymes in the synthesis pathway, thus improving the reaction flux and reducing cross-reactions [11,18,19]. Therefore, a variety of rational enzyme assembly or colocalization strategies have been developed to synthesize high-value products [12,13,18,20,21,22]. These engineered systems range from simple fusion proteins to scaffold-free self-assemblies, synthetic scaffolds, or physical compartments [23,24,25,26,27,28].

As the first attempt to co-localize different enzymes, direct fusion of metabolic enzymes by a short linker sequence to catalyze subsequent reactions has been shown to improve pathway flux in multiple circumstances [23,29,30]. At first, in order to study the properties of artificial bifunctional enzymes, Bulow et al. fusion-expressed the structural genes for β-galactosidase (lacZ) and galactokinase (galK) of *Escherichia coli* in vitro by connecting the *lacZ* gene to the N-terminus of the *galK* gene, and the fusion protein exhibited the enzymatic activity of both lacZ and galK [31]. Then, using a similar approach, Meynial Salles et al. constructed a strain that can produce glycerol efficiently with glucose as a substrate by fusing the enzymes of a *Saccharomyces cerevisiae* glycerol pathway together in *E. coli* [29]. Although enzyme direct fusion can effectively achieve the aggregation of enzymes, and is easy to operate in gene engineering, it still has many shortcomings. First, the direct fusion of two enzymes often results in a decrease in enzyme activity of one or both of the enzymes. Second, this method is not suitable for more than two reactions, nor can it directly regulate the stoichiometric ratio of enzymes [23]. Therefore, direct enzyme fusion is rarely used nowadays; instead, the commonly used strategies are the more advantageous multi-enzyme complex construction, enzyme assembly, and pathway sequestration, which can be realized by using artificial protein scaffolds or physical compartments [8,11,32].

In this review, using some specific examples, we provide an update on the current evidence and scope of enzyme assembly strategies in the process of microbial metabolism, and we systematically summarize the latest progress achieved in various assembly approaches. Moreover, we discuss the challenges and application prospects of enzyme assembly in the field of synthetic biology and metabolic engineering.

## 2. Scaffoldless Engineered Enzyme Assembly

### 2.1. Interaction Pair or Affinity Peptide Guided Enzyme Assembly

Many protein–protein interaction domains or short peptides of scaffolds, adapters, and signaling proteins can fold independently and can retain their binding function when recombined to the N-/C-terminus or even the interior of another protein [33,34,35,36,37]. By combining the heterogeneous high-affinity interaction of the protein domain/peptide ligand and the enzyme property of homologous oligomerization, a strategy for scaffold-free self-assembly of multiple oligomeric enzymes has been proposed and verified, both in vitro and in vivo [38]. Selecting octameric leucine dehydrogenase (LDH) and dimeric formate dehydrogenase (FDH) [39] as a technologically relevant model and the PDZ (PSD95/DlgA/Zo-1 domain from the adaptor protein syntrophin) domain and corresponding ligand (PDZlig) [34,40] as the LDH and FDH interaction interfaces, the cofactor recycling frequency was increased from 516 to 1007 h^−1^ at 10 μm of NAD^+^, and the conversion ratio for the first hour of the reaction was increased by approximately two times compared with a control with equal amounts of unassembled LDH and FDH [38]. A similar strategy, which organizes methanol dehydrogenase, 3-hexulose-6-phosphate synthase, and 6-phospho-3-hexuloseisomerase into an engineered supramolecular enzyme complex using an SH3–ligand interaction pair, was used to enhance the conversion of methanol to fructose-6-phosphate [41]. In combination with the utilization of an “NADH Sink” to prevent reversible formaldehyde reduction, it was found that compared with unassembled enzymes, fructose-6-phosphate production was increased 97-fold in vitro and nine times in vivo, respectively [41]. In addition, a method for the precise and reversible inducible self-assembly of dodecamer nitrilase in vivo (in *E. coli*) and in vitro (in a cell-free solution) was developed by means of the photoswitch-improved, light-inducible dimer system, which can induce protein–protein dimerization, and the assemblies were shown to retain 90% of the initial activity of nitrilase in vitro and in vivo. They even could be reused at least four times in vitro with 90% activity [42]. However, it is not difficult to see that the above-mentioned enzyme assembly requires a higher polymerization level of the enzyme itself, thereby reducing the universality of the strategy.

**Figure 1:**
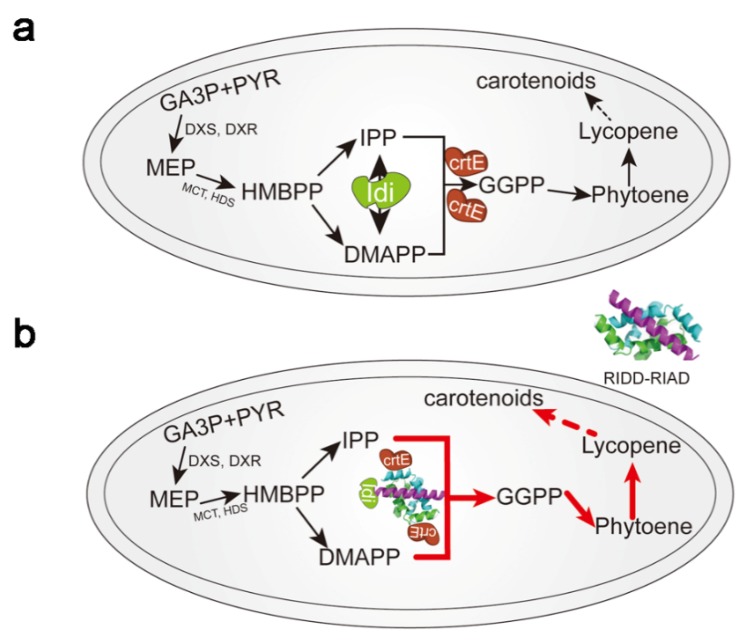
Schematic diagram showing Idi–CrtE (isopentenyl diphosphate isomerase-Geranylgeranyl diphosphate synthase) enzyme assembly. (**a**) Pathway of carotenoid synthesis in bacterial cells. (**b**) Idi–CrtE enzyme assembly using the RIAD–RIDD peptide enhances carotenoid synthesis. For carotenoid synthesis, Idi and CrtE represent the limiting step between the upstream MEP pathway and the downstream carotenoid pathway. GA3P: D-glyceraldehyde 3-phosphate; PYR: pyruvate; MEP: 2-C-methyl-D-Erythritol-4phosphate; HMBPP: 1-hydroxy-2-methyl-2-(E)butenyl4-diphosphate; DXS: 1-deoxyxylulose-5-phosphate synthase; DXR: 1-deoxy-D-xylulose-5-phosphate reductoisomerase; MCT: 4-diphosphocytidyl-2-C-methyl-Derythritol synthase; HDS: 1-hydroxy-2-methyl-2-(E)-butenyl 4-diphosphate synthase; IPP: isopentenyl pyrophosphate; DMAPP: dimethylallyl pyrophosphate; GGPP: geranylgeranyl pyrophosphate.

Kang et al. created scaffold-free enzyme assemblies by using a pair of short peptide tags RIAD and RIDD [28], which originate from the cAMP-dependent protein kinase (PKA) and the A kinase-anchoring proteins (AKAPs), respectively [43,44,45]. RIDD refers to a docking and dimerization domain of the R subunits of PKAs, and the RIAD peptide is derived from an amphipathic helix of the anchor domain of an AKAP that specifically binds to the RIDD dimer [46,47]. Further analysis showed that with RIAD–RIDD interaction, the assembly of enzymes for the menaquinone biosynthetic pathway yielded protein nanoparticles with varying stoichiometries, geometries, sizes, and catalytic efficiencies in vitro. In addition, a similar strategy was used to assemble the last enzyme of the mevalonate pathway with the first enzyme of the carotenoid pathway (Figure 1), resulting in an increase in carotenoid production by 5.7-fold in *E. coli* [28]. In general, Kang et al. proposed a simple metabolic control strategy, which provides a new direction for scaffold-free enzyme assembly; however, when the target enzyme is a monomer rather than an oligomer, the RIAD–RIDD trimer in the cell can only aggregate up to three enzymes (two kinds of enzymes). This may not be sufficient for some types of biosynthesis pathways, so the application scope of this strategy may have some limitations.

### 2.2. Enzyme Aggregation Guided by Active Inclusion Bodies

Traditionally, inclusion bodies (IBs) were thought to be commonly formed during the overexpression of heterologous proteins, and were regarded as waste repositories for unfolded or misfolded proteins that lack biological function. However, as early as 1989, Worrall and Goss found that IBs of beta-galactosidase contain correctly folded proteins, and the enzyme activity in the IBs is one-third that of soluble galactosidase [48]. In recent years, catalytically active inclusion bodies (CatIBs) formed by fusion of the target enzyme and the coiled-coil domain or aggregation-prone protein have been proposed [49,50,51,52,53,54]. CatIBs represent a promising new form of carrier-free enzyme immobilization, and might have important implications for protein aggregation in vitro or in vivo. For instance, Arié et al. found that when the soluble periplasmic enzymes alkaline phosphatase and β-lactamase fused with the C-terminus of MalE31, an aggregation-prone variant of maltose-binding protein in *E. coli*, the fusion proteins could accumulate in IBs in an insoluble form, but the enzyme activity could still be retained inside periplasmic IBs [52]. Taking the target enzymes of lipase A from *Bacillus subtilis*, hydroxynitrile lyase from *Arabidopsis thaliana*, and 2-succinyl-5-enolpyruvyl-6-hydroxy-3-cyclohexene-1-carboxylate synthase (MenD) from *E. coli* as examples, by expressing a fusion gene composed of a coiled-coil domain and a target enzyme, CatIBs were successfully produced and were used in aqueous and micro-aqueous organic-solvent-based reaction systems with excellent stability and recyclability [49]. In addition, by using CatIBs for the synthesis of (R)-benzoins, Kloss et al. found that the activity was three times higher than that of soluble enzymes [55].

Recently, our research group performed a preliminary study on the biological function of CatIBs in vivo. When investigating the properties of the stomatin/prohibitin/flotillin/HflK/C (SPFH) domain of the HflK protein, we found that the SPFH domains could promote the formation of functional IBs, and these functional IBs could obviously improve enzymatic catalysis in vivo, as proven by the examples with whole-cell biocatalysis of phenyllactic acid by *E. coli* and the biosynthesis of N-acetylglucosamine by *B. subtilis* [56]. Bimolecular fluorescent complimentary (BiFC) and Förster resonance energy transfer (FRET) analysis showed that the mechanism of this effect may be that the formation of CatIBs can promote intermolecular polymerization, which is consistent with what was speculated by Arié et al. [52]. However, the study about the mechanism of CatIBs enhancing catalytic efficiency, especially in vivo, is just in the initial stage, and further studies are needed to expand the knowledge on in vivo biocatalytic applications of CatIBs.

## 3. Enhancing Multi-Enzyme Biosynthesis Using Synthetic Scaffolds

### 3.1. Nucleic Acid Scaffold

With the development of nucleic acid nanotechnology and computer simulation technology, the construction of synthetic scaffolds based on nucleic acid molecules to assemble pathway enzymes has been gradually developed [57,58,59,60,61]. Nucleic acids can be selected as scaffolds depending on their own characteristics. For example, the interaction between DNA/RNA and proteins can be achieved by hybridization (DNA–DNA/DNA–RNA/RNA–RNA) or by using the DNA binding domain of the zinc-finger protein (ZFP) or transcription activator-like effector (TALE) [62,63]. In addition, short chains of nucleic acid molecules can be folded into various structures or assembled into dimer or polymer components to synthesize various three-dimensional structures with specific programmability [64,65,66,67].

To date, due to the DNA–DNA binding or DNA–protein binding specificity, DNA architectures have been investigated as another potential molecular building platform for organizing enzymes, and potential applications have begun to emerge [8,64,68,69,70,71,72,73]. For example, Müller and Niemeyer used the DNA-directed assembly of covalent conjugates of DNA oligonucleotides and glucose oxidase or horseradish peroxidase to generate supramolecular complexes, in which the two enzymes were arranged with defined spatial orientation. Combined with the kinetic measurements, they revealed a significant increase in the reactivity of the complexes, in which glucose oxidase and horseradish peroxidase were immobilized in direct proximity on a complementary DNA carrier [74]. When glucose oxidase and horseradish peroxidase were covalently linked to short DNA oligonucleotides that can specifically hybridize onto polyhexagonal DNA nanostructures, a significant increase in product formation was found after the enzyme spacing was shortened from the original four hexagons (~33 nm apart) to two hexagons (~13 nm apart) [68]. The reason for the improved catalytic efficiency was not only that the concentrations of the local intermediates increased due to the shortened enzyme spacing, but also that the diffusion of the intermediates on the enzyme surface was limited [68,75]. In addition to the above-mentioned DNA scaffolds, other kinds of supramolecular DNA scaffolds or DNA origami that can be used to mediate the multi-enzyme cascade reaction have been found [76,77,78,79]. By using the programmability of DNA nanostructures, we can adjust the distance, position, and stoichiometric number of enzymes to explore the best effect of DNA scaffolds [80]; however, the application of this strategy in vivo is challenging, because the concentrations of single-stranded nucleic acid building blocks and environmental properties important for nucleic acid folding (e.g., temperature, ions) are not easily manipulated in living cells [57]. In addition, the chemical modification technology for covalently linking specific oligonucleotide sequences with lysine residues of pathway enzymes in vitro is expensive, and may affect enzyme activity, resulting in these strategies having reduced feasibility and universality.

**Figure 2:**
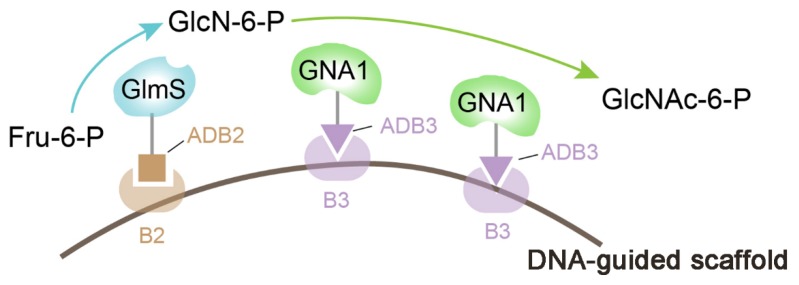
Schematic diagram of the spatial organization of GlmS and GNA1. The zinc finger protein ADB2 (corresponding to B2 binding sequence) was fused with GlmS, and ADB3 (corresponding to B3 binding sequence) was fused with GNA1. GlmS: glucosamine-6-phosphate synthase; GNA1: GlcNAc (N-acetylglucosamine)-6-phosphate N-acetyltransferase; Fru-6-P: fructose-6-phosphate; GlcN-6-P: glucosamine-6-phosphate; GlcNAc-6-P: N-acetylglucosamine-6-phosphate.

Determining how to apply the in vitro model of DNA scaffolds to the internal environment is a new challenge for researchers. So far, researchers have begun to use the characteristics of specific bindings of nucleic acid binding proteins (such as ZFPs and TALEs) and nucleotide sequences to realize the assembly of pathway enzymes [81,82,83,84,85,86,87]. For example, through fusing glucosamine-6-phosphate synthase (GlmS) and GlcNAc (N-acetylglucosamine)-6-phosphate N-acetyltransferase (GNA1) with the zinc finger proteins ADB3 and ADB2 by pHT plasmid, respectively, Liu and co-workers successfully constructed a DNA-guided scaffold system to modulate the activities of GlmS and GNA1 (Figure 2), and they found that the co-location and control of the stoichiometric ratios of GlmS and GNA1 significantly increased the GlcNAc titer from 1.83 to 4.55 g/L in a shake flask [84]. Although plasmid DNA scaffolds have some obvious advantages, such as having the ability to accommodate multiple interacting motifs and variable spacing, these scaffolds need multiple ZFP domains to modify the pathway enzymes, and the maximum concentration of DNA scaffolds in cells is limited by the plasmid copy number.

As an information-bearing molecule, RNA can form elaborate and dynamic structures via base pairing. Due to having a good understanding of the thermodynamics of nucleic acid base pairing, especially in relation to protein folding, researchers have regarded RNA as a natural target for bioengineering [88,89,90], and a variety of RNA structures have been constructed for multiple purposes in metabolic engineering, such as the use of RNA nanostructures for organizing biomolecules [91,92,93]. The self-assembly of RNA nanostructures depends on the RNA-specific sequence motifs, such as base pairing between hairpin stems (kissing loops) and chemical modifications for localizing enzymes on a structure, both of which require annealing at nonphysiological Mg^2+^ concentrations (10–15 mM) [25]. Therefore, this strategy cannot be implemented in vivo. To apply this technology in vivo, Delebecque et al. designed and assembled discrete one- or two-dimensional RNA scaffolds with distinct protein-docking sites for the spatial organization of a hydrogen-producing pathway [94]. Similar RNA scaffolds were also used to optimize the pentadecane production in *E. coli* by heterologous expression of acyl-ACP reductase and aldehyde deformylating oxygenase [95]. Unfortunately, the self-assembly of discrete RNA structures with complex geometry in vivo is still difficult to achieve.

### 3.2. Protein Scaffolds

The protein scaffold strategy refers to an enzyme assembly approach that uses protein–protein interaction domains to fix pathway enzymes on a protein scaffold to enhance metabolic pathway flux [11]. This strategy can balance the stoichiometry of related pathway enzymes by regulating the proportion and order of receptor domains [96]. One of the key tasks in constructing the whole protein scaffold system is to select protein–protein interaction domains and ligands. The structural modularity of the protein–protein interaction domains is crucial, because they need to maintain binding activity in a non-native environment of translational fusion [97]. In the existing reports, many modular protein–protein interaction domains have been characterized and employed, such as the SH3 (Src homology 3 domain from the adaptor protein CRK) domain [98], the PDZ (PSD95/DlgA/Zo-1 domain from the adaptor protein syntrophin) domain [40,99], the GBD (GTPase binding domain from the actin polymerization switch N-WASP) domain [100], leucine zippers [101], the PhyB/Pif3 (phytochrome B/phytochrome interacting factor 3) light switchable binding domains [102], and even affibody molecules (58-residue, non-immunoglobulin affinity proteins derived from the Fc-binding domain of *Staphylococcus aureus* protein A) [103]. A successful example of using an intracellular protein scaffold for enhancing metabolic flux is the (GBD)_x_–(SH3)_y_–(PDZ)_z_ protein scaffold constructed by fusion expression of the above interaction domains GBD, SH3, and PDZ [96]. Utilizing the (GBD)_x_–(SH3)_y_–(PDZ)_z_ protein scaffold, multi-enzyme complexes of the mevalonate biosynthetic pathway consisting of acetoacetyl-CoA transferase (atoB), hydroxy-methylglutaryl-CoA synthase (HMGS), and hydroxymethylglutaryl-CoA reductase (HMGR) were constructed, and the stoichiometric number of enzymes was optimized by adjusting the proportion and order of protein scaffolds, resulting in the mevalonate titer being 77-fold higher than that without enzyme co-localization [96]. It is worth noting that there was only one SH3 domain difference between the (GBD)1–(SH3)2–(PDZ)2 protein scaffold and the (GBD)1–(SH3)1–(PDZ)2 protein scaffold, yet the titer of mevalonate increased by 77 and 4 times, respectively, indicating that the precise control of stoichiometric ratios is likely to be a key point to break the bottleneck of product synthesis. This protein scaffold has also been successfully applied in other biosynthesis pathways, such as the glucaric acid synthesis pathway and the resveratrol synthesis pathway [16,96,104,105,106]. Although the exact mechanism by which protein scaffolds increase the metabolic flux is not clear, it was speculated that the interaction domain and enzyme oligomerization promoted a large number of enzymes to form a large complex, and the intermediate metabolites of the synthesis pathway were consumed by the enzymes in the complex before diffusion [8]. However, for some metabolic pathways, the improvement effect was not significant [24], most likely due to this design lacking an inherent organized structure, meaning that it may aggregate in unpredictable ways, thereby eventually hindering the development of a rational design process [8,32].

**Figure 3:**
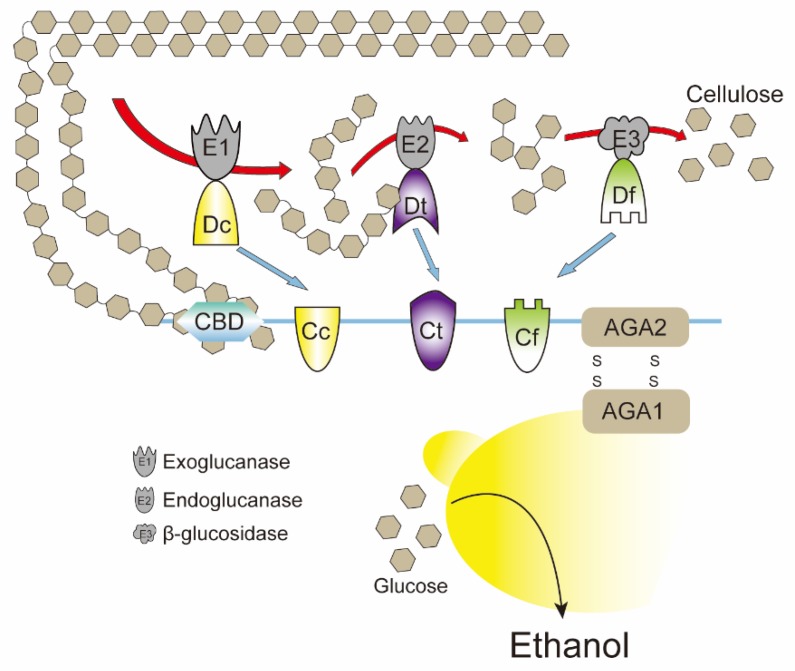
Schematic of minicellulosome assembly on the cell surface. A trifunctional scaffoldin consisting of an internal CBD (cellulose binding domain) flanked by three divergent cohesion domains is displayed on the yeast cell surface. *E. coli* cell lysates containing endoglucanase, exoglucanase, and beta-glucosidase fused with corresponding dockerin domains are mixed with engineering yeast cells for minicellulosome assembly. AGA1/2: GPI anchor; Cc: cohesion domain from *C. cellulolyticum*; Dc: dockerin domain from *C. cellulolyticum*; Ct: cohesion domain from *C. thermocellum;* Dt: dockerin domain from *C. thermocellum;* Cf: cohesion domain from *R. flavefaciens*; Df: dockerin domain from *R. flavefaciens.*

In addition to constructing protein scaffolds in cells, the protein scaffold system can also be constructed outside the cell, and the construction of extracellular protein scaffolds often requires the assistance of the plasma membrane [107,108,109,110]. The cellulosome complex of anaerobic cellulolytic bacteria is a typical natural example of using protein scaffolds to assemble enzymes to enhance the synergistic activity among different resident enzymes [111,112]. In this cellulosome complex, via the cohesion–dockerin interaction, a non-catalytic subunit called scaffoldin can secure the enzymatic subunits into this complex [111]. Inspired by this natural protein scaffold, Tsai et al. successfully assembled the multi-enzyme cascade reaction of endoglucanase, exoglucanase, and beta-glucosidase on the surface of *Saccharomyces cerevisiae* [108]. As shown in Figure 3, a scaffoldin consisting of an internal cellulose binding domain and three divergent cohesin domains from *Ruminococcus flavefaciens, Clostridium cellulolyticum*, and *Clostridium thermocellum* were immobilized to yeast cells by a glycosylphosphatidylinositol (GPI) anchor. Then, endoglucanase, exoglucanase, and beta-glucosidase fused with the corresponding dockerin domain were expressed in *E. coli*, and the cell lysates containing these cellulases were mixed with the above engineering yeast cells for the functional assembly of the minicellulosome. The final results showed that the obtained multi-enzyme cascade reaction realized the combination of cellulose hydrolysis and ethanol production, and the ethanol concentration was more than 2.6 times higher than that achieved by adding the same amount of free purified cellulase [108]. However, the design and construction of extracellular membrane protein scaffolds is more complex, and due to the complexity and instability of the extracellular microenvironment, the current application of this strategy is still limited.

### 3.3. Lipid-Containing Scaffolds

**Figure 4:**
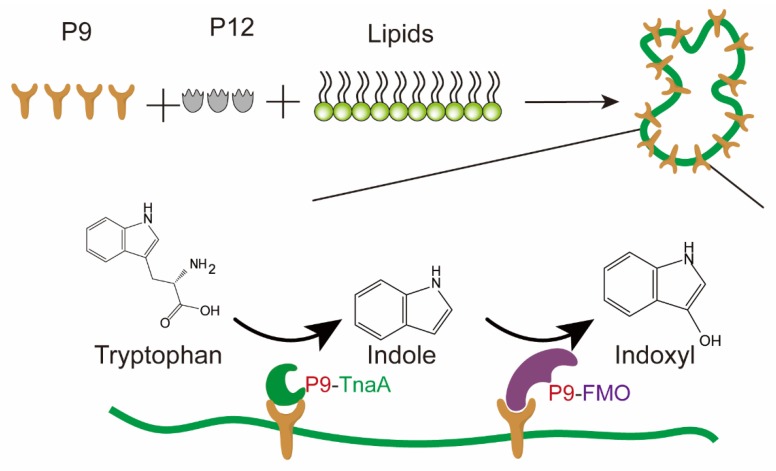
Schematic diagram of enzyme assembly by using a lipid-containing scaffold. Indigo production was chosen as an example to show the fixation mode between a lipid-containing scaffold and enzymes (tryptophanase TnaA and flavin-dependent monooxygenase FMO). Both TnaA and FMO were fused with P9.

In addition to the nucleic acid scaffold and protein scaffold mentioned above, in recent years, researchers have proposed another synthetic scaffold called the lipid-containing scaffold [88]. Lipids are likely to be unique scaffold building materials that can be used as the anchors of membrane proteins. Also, lipids can form membrane barriers that allow small molecules to selectively enter and exit compartments [88]. For example, in yeast, the synthetic protein scaffolds for the ethyl acetate biosynthetic pathway can be located on lipid droplet membranes [113]. However, the construction of a lipid-containing scaffold requires the co-assembly of lipids and related proteins. Recently, it was reported that the expression of the viral proteins P8, P9, and P12 of Bacteriophage ϕ6 in *E. coli* can form circular particles containing a mixture of lipids and proteins [114]. Inspired by this finding, Myhrvold et al. successfully built synthetic lipid-containing scaffolds in *E. coli* using two ϕ6 proteins P9 and P12, and found that this scaffold system can significantly enhance indigo production by immobilizing two enzymes (tryptophanase TnaA and flavin-dependent monooxygenase FMO) involved in indigo biosynthesis (Figure 4) [88]. As a newly developed synthetic scaffold, there are still many gaps in the research of lipid-containing scaffolds, especially regarding the mechanism analysis and application of diversified products. However, in view of the lipid components, lipid-containing scaffolds may have their special advantages in the synthesis of steroids, fatty acids, and other lipid products.

## 4. Physical Compartments for Pathway Sequestration

Another effective approach for metabolic pathway flux enhancement is to physically encapsulate pathway enzymes into distinct compartments. The compartmentalized pathway can benefit from physical barriers that block the exchange of metabolites and protect heterologous enzymes from adverse interactions within host cells [8]. In addition, compartmentalization features the unique advantages of physical sequestration of limiting toxic intermediates and providing a segregated environment for some specific reactions [115,116].

### 4.1. Eukaryotic Physical Compartments

**Figure 5:**
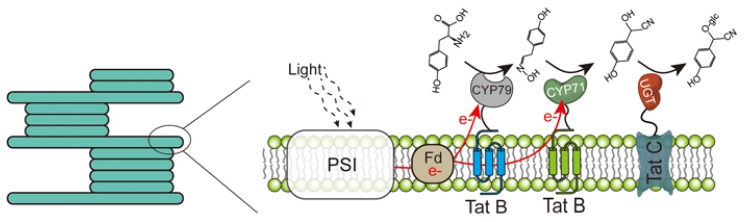
Schematic diagram of the Tat-scaffolded dhurrin pathway. Two P450s (CYP79A1, CYP71E1) and a soluble UDP-glucosyl transferase (UGT85B1) are anchored in the thylakoid membrane by exchanging the membrane anchors with the components of the self-assembled Tat B and Tat C.

The organelles with membrane structures in eukaryotes provide a potential target for improving the biosynthesis efficiency [113]. Many secondary metabolic pathways in plants that produce valuable pharmaceuticals, such as alkaloids, are usually organized on the endoplasmic reticulum and vacuoles, and the key steps in long-chain fatty acid synthesis and lipid storage in yeast are also localized to the endoplasmic reticulum [117,118,119]. Now, more and more studies are being conducted on the artificial relocation of pathway enzymes. In addition to the endoplasmic reticulum and vacuole mentioned above, compartments of enzyme complexes can also be peroxisomes, chloroplasts, mitochondria, and the Golgi apparatus [120,121]. Dhurrin (D-glucopyranosyloxy-(S)-p-hydroxymandelonitrile) is a cyanogenic glucoside, and its synthetic pathway consists of two endoplasmic reticulum membrane-bound cytochrome P450 enzymes (CYP79A1 and CYP71E1) and a soluble UDP-glucosyltransferase (UGT85B1) [122,123]. Among them, the cytochrome P450 enzymes require an NADPH-dependent reductase to provide electrons to give reducing power [123,124]. The ferredoxin in chloroplasts can provide a large number of electrons for P450 through the photosynthetic electron transport chain, so relocating the dhurrin biosynthesis pathway to chloroplasts can successfully synthesize dhurrin without expressing NADPH-dependent reductase [125]. Further co-localization of these dhurrin pathway enzymes in the thylakoid membrane results in a significant five-fold increase in product formation, accompanied by a decrease in off-pathway intermediates, by exchanging the membrane anchors of the enzyme to the components of the self-assembled, twin-arginine translocation pathway (Figure 5) [126]. Besides, Avalos et al. found that compartmentalization of the Ehrlich pathway into yeast mitochondria can dramatically improve the production of branched chain alcohols (isobutanol) compared with expression of the same pathways in the cytoplasm [127]. Further analysis showed that targeting these enzymes to mitochondria can lead to higher local enzyme concentrations and can limit the concentration of intermediates in mitochondria, avoiding the loss of intermediates to competition pathways [127].

### 4.2. Protein-Based Compartments in Prokaryotic Cells

Unlike eukaryotic cells, prokaryotic cells, such as bacteria, do not possess the various complex organelles with membrane structures for use as compartments; however, the interiors of bacteria can contain various protein-based compartments, the most typical of which are bacterial microcompartments (BMCs). BMCs are a family of 80–200 nm proteinaceous polyhedrals that can be seen as organelle-like structures with an outer semipermeable scaffold [8,27,128,129,130]. So far, the BMCs used for encapsulating metabolic pathways for improved production of heterologous metabolites have been emphasized in many studies [27,131,132,133,134,135,136,137,138,139,140,141,142]. Numerous crystal structure studies and comparative analyses have revealed that the signature domain of BMCs with different functions and distant relatives has little structural variation, indicating that the signature domain plays a key role in the assembly of the BMC shell [143,144]. The main constituents of BMC shells are typically small (∼100 amino acids) proteins containing the BMC domain (BMC-H) [145,146]. In vitro, the purified BMC-H proteins can form distinct architectures, mainly including spheroids, extended nanotubes, and honeycombed tiles [147,148,149,150]. Also, overexpression of BMC-H homologs, such as PduA, MicH, RmmH, and CcmK2 in vivo can also form a variety of high-order structures through self-assembly, such as tubes, filaments, and other structures [27,151,152,153,154,155,156].

There are different strategies for organizing enzymes in BMC-H assemblies. A more direct method for heterologous assembly is to use binding motif BMCs that are natively employed to recruit cargo [157,158]. BMC core proteins usually contain small peptides (about 20 amino acids) called “encapsulation peptides” as extensions of N- or C- termini [27,132,136,141,159]. These encapsulated peptides are characterized by the formation of an amphiphilic α-helix that can interact with the shell protein [158], and Jakobson et al. demonstrated that non-native encapsulated peptides can interact with non-cognate BMCs [159]. For example, through fusing the encapsulated peptides with the pyruvate decarboxylase and alcohol dehydrogenase to direct the two enzymes to empty BMCs, a PDU BMC was engineered into an ethanol reactor, and the strains containing the redesigned BMCs produced elevated levels of alcohol [133]. However, due to the uncertainty in the affinity encapsulated peptide–BMC component binding and interface location [133,157,158], the application of encapsulated peptides for predictive recruitment is still limited.

Another strategy is to append natural or synthetically derived protein–protein interaction domains to BMC-H proteins. However, whether the fusion of interaction domains to BMC-H proteins will affect higher-order self-assembly must be determined. In previous studies, it was shown that whether BMC-H proteins are fused with a small affinity tag or with a larger fluorescent protein, the modified BMC-H protein can still incorporate into BMCs normally through self-assembly [134,160,161,162,163]. However, Cameron et al. pointed out that the premise of the fusion of fluorescent proteins and the main shell protein Ccmk2 to form functional BMCs is that there must be unmodified Ccmk2 proteins [163]. In recent years, researchers have applied this strategy to pathway enzyme assembly. For example, as a shell protein from the *Citrobacter freundii* Pdu BMC, PduA itself can form a tile by hexamerization, and then it assembles into the facets of the BMC shell [143,164]. By modifying the C-terminus of PduA, PduA* with improved solubility was obtained, and when PduA* was overexpressed in *E. coli*, hollow filaments with diameters of about 20 nm were formed, presenting tractable scaffolds for tethering pathway enzymes [152,154]. Based on these previous research results, Warren and Woolfson’s group established a three-component system comprising PduA* and two complementary coiled-coil peptides, and found that tethering metabolic enzymes for ethanol production to the PduA* scaffold by coiled-coil peptides increased the local and relative concentrations, ultimately significantly improving ethanol production [27].

## 5. Perspectives and Conclusions

In this review, we have summarized and discussed different strategies for spatially assembling metabolic pathways, including scaffold-free enzyme assembly, nucleic acid scaffolds, protein scaffolds, lipid-containing scaffolds, and physical compartments (Table 1). Enzyme assembly has attracted a wide range of interest in the fields of synthetic biology and metabolic engineering; however, some problems and controversies still exist.

It is known that metabolic flux is regulated by enzyme kinetics and substrate diffusion, and macromolecular crowding in cells can promote enzyme associations [121,165,166]. Moreover, the fluidity in the cytoplasm and in vivo environments obviously affects the metabolic activity and enzyme kinetics [167,168], while an effective enzyme assembly could pass the product of one reaction site to an adjacent site to prevent metabolic intermediates from diffusing away [121]. However, such a mechanism, known as proximity channeling, is not universally recognized. For example, some researchers have proposed that simply fusing the two enzymes would not cause productive channeling by calculating the distance between substrates and enzymes as well as the distance between the active sites of two adjacent enzymes [169]. By developing a quantitative model, Castellana et al. proposed that assembling multiple enzymes together to form compact clumps can not only accelerate the processing of intermediates through metabolic channeling, but can also regulate the flux distribution, especially at the metabolic branch point [169]. In general, the more widely accepted explanation for the increased productivity of enzyme assembly is that enzyme assembly can increase the local concentration of pathway metabolites and enzymes, and reduce the possibility of unintentional interactions with other cell components.

Some scholars propose that on the length scale of bacteria, diffusion is sufficiently fast to supply ample substrate for enzymatic reactions without the need for metabolite channeling [170]. Biochemical experiments and analysis reveal that the rate of most enzyme-catalyzed reactions is 1000–10,000 times slower than the diffusion rate for collisions of proteins and metabolites [171]. However, the kinetic parameters measured in vitro cannot represent enzyme activity in vivo. In fact, the cytoplasm of bacteria is filled with non-uniform macromolecules and “binding” water, so the viscosity of the intracellular suspension is very high, approaching the characteristics of glass-forming liquid [167]. According to Stokes–Einstein law, such a high viscosity can cause transport obstacles [121]. Therefore, spatial orientation of enzyme complexes is still necessary for small bacteria. Another point that has attracted wide attention is whether the introduction of exogenous affinity peptides or scaffold structures in enzyme assembly strategies will cause a metabolic burden to cells. For example, strains over-expressing PduA* alone can generate filamentous structures spanning the length of the cell, but the cell division is inhibited [27]. Kafri et al. pointed out that when exogenous proteins make up 30% of the intracellular proteome, the cell burden obviously increases, and intracellular protein toxicity can be observed [172,173]. Considering that the biosynthesis of bio-based products often requires the introduction of exogenous enzymes or the overexpression of enzymes, due to their low activity, the selection and introduction of exogenous affinity peptides or scaffold structures need special attention from researchers.

The core technology of enzyme assembly strategies is to fix the target enzymes in/on the scaffold structure in an effective, directional, orderly, and stable manner, or to promote the target enzymes to gather closely in a controllable microdomain. Although these strategies have achieved some success, the challenge is still huge for the efficient assembly of enzymes.

First, most of the current enzyme assembly strategies can only be used for simple metabolic pathways or for the assembly of 2–3 enzymes, which is difficult to apply to the systematic enzyme assembly of complex metabolic pathways, such as the glycolysis pathway or the synthesis pathway of menaquinone-7. The application of short peptide tags provides an opportunity to overcome this challenge; this has been used in the menaquinone biosynthetic pathway to assemble enzyme pathways through short peptide tag interactions, in order to generate protein nanoparticles with various stoichiometries, geometries, and enzyme pathway sizes. Second, affinity peptides are generally polar, and the fusion expression of polar peptides at the N-/C-terminus of the target enzyme often affects the enzyme activity. There are many natural multi-enzyme complexes in nature—for example, enzymes catalyzing the last two steps of tryptophan synthesis form a complex where the intermediate indole is channeled from one active site to the other [174,175,176,177]. Understanding the mechanism of the formation and function of these complexes will help improve the existing strategies of enzyme assembly, and could even lead to the development of new strategies. Third, both the organelle-like compartments in eukaryotic cells and the protein-based compartments in prokaryotic cells may be barriers for pathway enzymes, products, and substrates in or out of the compartments. Specifically, with the advent of synthetic biology and advances in protein engineering, designing proteins with elaborate control points and reactions that are not currently known in nature is now a possibility. Thus, it is possible to perform enzyme engineering to obtain artificial BMC protein shells with the ability to selectively facilitate the entry or exit of substrates and products.

In general, future investigations of enzyme assembly strategies should be focused on signal peptide screening, enzyme engineering, and membrane modification. Signal peptide screening considerations should be targeted at relieving polar toxicity to enzymes and promoting the localization of enzymes. Enzyme engineering considerations should target the protein-based compartments in prokaryotic cells, in order to facilitate the entry or exit of substrates and products. Membrane modification should target enhancing cell tolerance to the microenvironment.

## Figures and Tables

**Table 1:** Application of different enzyme assembly strategies in vivo.

Enzyme Assembly Strategy		Number of Immobilized Enzymes	Reference
Scaffold-free enzyme assembly	Interaction pair or affinity peptide guided enzyme assembly	2–3	[28,38,41]
	CatIBs	1–2	[56]
Nucleic acid scaffold	DNA scaffold	2	[84]
	RNA scaffold	2–4	[95]
Protein scaffold	(GBD)_x_–(SH3)_y_–(PDZ)_z_ protein scaffold	3	[96]
	Protein scaffold outside the cell	3	[108]
Lipid-containing scaffold		2	[88]
Physical compartment	Eukaryotic physical compartments	3	[125,126]
	Protein-based compartments in prokaryotic cells	2	[27]
